# Analysis of the Potential for *N*^4^-Hydroxycytidine To Inhibit Mitochondrial Replication and Function

**DOI:** 10.1128/AAC.01719-19

**Published:** 2020-01-27

**Authors:** Zachary M. Sticher, Gaofei Lu, Deborah G. Mitchell, Joshua Marlow, Levi Moellering, Gregory R. Bluemling, David B. Guthrie, Michael G. Natchus, George R. Painter, Alexander A. Kolykhalov

**Affiliations:** a Emory Institute for Drug Development, Emory University, Atlanta, Georgia, USA; b Drug Innovation Ventures at Emory, Atlanta, Georgia, USA; c Department of Pharmacology and Chemical Biology, Emory University School of Medicine, Atlanta, Georgia, USA

**Keywords:** *N*^4^-hydroxycytidine, NHC, POLRMT, mitochondrial toxicity, ribonucleoside analog

## Abstract

*N*^4^-Hydroxycytidine (NHC) is an antiviral ribonucleoside analog that acts as a competitive alternative substrate for virally encoded RNA-dependent RNA polymerases. It exhibits measurable levels of cytotoxicity, with 50% cytotoxic concentration values ranging from 7.5 μM in CEM cells and up to >100 μM in other cell lines. The mitochondrial DNA-dependent RNA polymerase (POLRMT) has been shown to incorporate some nucleotide analogs into mitochondrial RNAs, resulting in substantial mitochondrial toxicity. NHC was tested in multiple assays intended to determine its potential to cause mitochondrial toxicity. NHC showed similar cytotoxicity in HepG2 cells incubated in a glucose-free and glucose-containing media, suggesting that NHC does not impair mitochondrial function in this cell line based on the Crabtree effect. We demonstrate that the 5′-triphosphate of NHC can be used by POLRMT for incorporation into nascent RNA chain but does not cause immediate chain termination. In PC-3 cells treated with NHC, the 50% inhibitory concentrations of mitochondrial protein expression inhibition were 2.7-fold lower than those for nuclear-encoded protein expression, but this effect did not result in selective mitochondrial toxicity. A 14-day incubation of HepG2 cells with NHC had no effect on mitochondrial DNA copy number or extracellular lactate levels. In CEM cells treated with NHC at 10 μM, a slight decrease (by ∼20%) in mitochondrial DNA copy number and a corresponding slight increase in extracellular lactate levels were detected, but these effects were not enhanced by an increase in NHC treatment concentration. In summary, the results indicate that mitochondrial impairment by NHC is not the main contributor to the compound’s observed cytotoxicity in these cell lines.

## Abstract (precis)

*N*^4^-Hydroxycytidine (NHC) is an antiviral ribonucleoside analog that acts as a competitive alternative substrate for virally encoded RNA-dependent RNA polymerases. It exhibits measurable levels of cytotoxicity, with 50% cytotoxic concentration values ranging from 7.5 μM in CEM cells and up to >100 μM in other cell lines.

## INTRODUCTION

Nucleoside analogs have played a critical role in antiviral therapy beginning with the approval of the first antiviral drug, idoxuridine, in 1963 for the treatment of herpes simplex virus (HSV) (1). Since then, nucleoside analogs have been approved for the treatment of infections caused by several DNA and RNA viruses responsible for significant morbidity and mortality, including human immunodeficiency virus (HIV), hepatitis B virus, hepatitis C virus (HCV), varicella-zoster virus, and human cytomegalovirus (2–6). The antiviral mechanism for these compounds requires that they be metabolized by intracellular kinases to their corresponding 5′-triphosphates, which can then serve as competitive substrates to be incorporated into the viral genome by virus-encoded polymerases. This genome incorporation results in cessation and/or perturbation of genome replication and/or gene expression.

Since phosphorylated ribonucleoside analogs mimic natural ribonucleotides, they may also be incorporated into human RNA by the mitochondrial DNA-dependent RNA polymerase (POLRMT) or by nuclear RNA polymerase (Pol) I, II, or III, resulting in undesired side effects (7, 8). The development of several ribonucleoside analogs has been stopped as a result of toxicity observed during clinical trials. BMS-986094, a prodrug of 2′-C-methylguanosine being investigated for the treatment of HCV, was halted during phase II clinical trials due to cardiac and kidney toxicity (9). Balapiravir, a prodrug of 4′-azidocytidine that was investigated for the treatment of HCV, was stopped in phase II clinical trials due to hematologic toxicity (10). In both cases, later studies suggested the cause of the observed toxicity was mitochondrial dysfunction (8). Zidovudine, the first approved antiretroviral for HIV treatment, was shown to have dose-limiting toxicity resulting from the depletion of mitochondrial DNA (mtDNA) (2). To examine early in the drug development process the potential for nucleoside analogs to cause mitochondrial toxicity, a series of *in vitro* assays have been developed to characterize the effects of a compound on mitochondrial DNA copy number and mitochondrial function. Key among these are the measurement of mtDNA levels, lactate production, and mitochondrial protein expression (7, 11–13).

*N*^4^-Hydroxycytidine (NHC) is a ribonucleoside analog currently in late-stage preclinical development. NHC has shown broad-spectrum antiviral activity against viruses in the *Togaviridae*, *Flaviviridae*, *Coronaviridae*, *Pneumoviridae*, and *Orthomyxoviridae* families (14–18). It has been shown that the 5′-triphosphate of NHC can serve as a competitive alternative substrate and be incorporated into viral RNA (14). Upon incorporation, NHC inhibits viral RNA genome replication, either by disrupting the secondary structure of the genome promoter regions and thus inhibiting replication of the virus (14) or by the introduction of mutations into the viral RNA genome that leads to error catastrophe (15, 19). Given that this compound is a ribonucleoside analog with the potential for interfering with mitochondrial function, a series of experiments designed to evaluate the effect of prolonged exposure to NHC on mitochondrial death or mitochondrial dysfunction *in vitro* was conducted.

## RESULTS

### Cytotoxicity of NHC in HepG2 and CEM cell lines.

Prior to conducting experiments on mitochondrial function, the 50% cytotoxic concentration (CC_50_) of NHC was determined in each of the cell lines used in this study. The results of these assays are summarized in Table 1 and in Table S1 in the supplemental material. The CC_50_ values for the human hepatic origin HepG2 cell line were measured in cells grown in glucose-containing media and in glucose-free media. Cells grown in the presence of glucose are subject to the Crabtree effect, whereby they are able to produce nearly all of their ATP through glycolysis, even though the cells possess functional mitochondria (20). Furthermore, it has been shown that cells are more susceptible to mitochondrial toxins when glucose is replaced with galactose in the incubation media (20). In a side-by-side experiment, the CC_50_ values for NHC were similar in HepG2 cells incubated with glucose and in HepG2 cells incubated with galactose instead of glucose. By this measure, NHC does not impair mitochondrial function in this cell line. Among other tested cell lines of different tissue and species origin, the T-lymphoblastoid origin CEM cell line was the most susceptible to NHC treatment, with CC_50_ values 3- to 4-fold lower than in other cell lines tested (see Table S1); therefore, the CEM cell line was chosen to evaluate the potential for NHC to cause mitochondrial toxicity. In addition, the HepG2 cell line was also chosen since HepG2 cells have been traditionally used to evaluate mitochondrial toxicity (2, 14, 20, 21) and have demonstrated their utility.

**TABLE 1:** Cytotoxicity of NHC in tissue culture

Cell line	CC_50_ (μM)
CEM	7.5
HepG2	42.3
HepG2/gal^*a*^	41.4
PC-3	267.1

a Cells were incubated in glucose-free medium containing galactose.

### NHC is efficiently converted in cells to its 5′-triphosphate.

**FIG 1:**
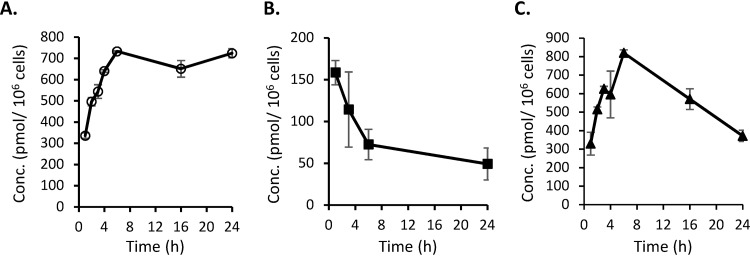
Time-concentration profiles of intracellular NHC–5′-triphosphate accumulation in cells incubated with NHC. (A) HepG2 cells incubated with NHC at 20 μM. (B) CEM cells incubated with NHC at 10 μM. (C) PC-3 cells incubated with NHC at 10 μM. The data are shown as means ± the standard deviations (SD) (*n* = 3).

Ribonucleoside analogs must be converted into their 5′-triphosphate metabolites to be utilized as the substrates by RNA polymerases and incorporated into RNA. To confirm that NHC is converted to its 5′-triphosphate form, NHC-TP, HepG2, PC-3, and CEM cells were incubated with NHC and intracellular levels of NHC-TP were quantified by liquid chromatography-tandem mass spectrometry (LC-MS/MS). In HepG2 cells treated with 20 μM NHC, intracellular NHC-TP levels reached a maximum concentration of 732.7 ± 2.9 pmol/10^6^ cells after a 6-h incubation (Fig. 1A) and remained stable for up to 24 h in the presence of drug in the media. This level appears to be higher than the previously reported value of 71.12 ± 22.66 pmol/10^6^ cells, which was determined after a 6-h incubation with 10 μM NHC (14). In CEM cells treated with 10 μM NHC, the intracellular levels of NHC-TP reached a maximum of 158.4 ± 14.5 pmol/10^6^ cells after a 1-h incubation (Fig. 1B). In PC-3 cells treated with 10 μM NHC, the intracellular levels of NHC-TP reached a maximum concentration of 819.5 ± 16.8 pmol/10^6^ cells after a 6-h incubation (Fig. 1C). These experiments confirm that NHC is efficiently converted to its 5′-triphosphate metabolite in all tested cells.

### NHC–5′-triphosphate is a substrate for POLRMT in primer extension assays.

**FIG 2:**
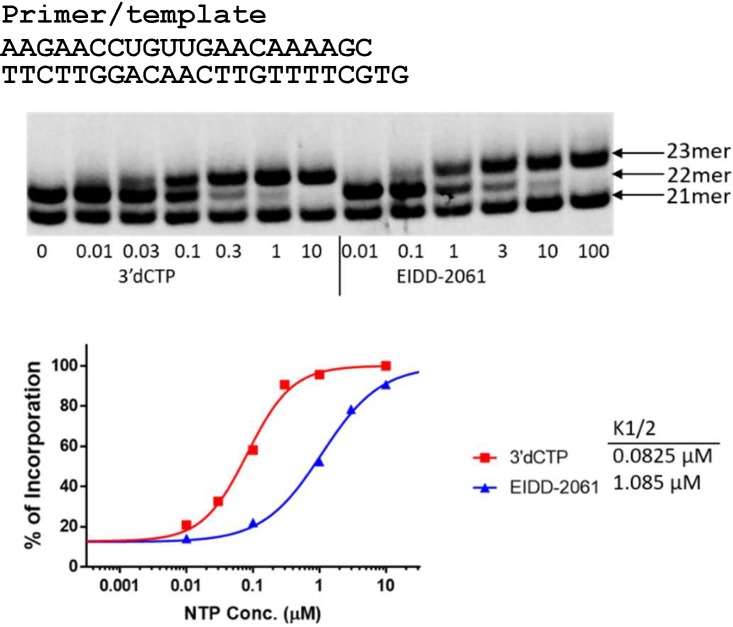
NHC-TP is incorporated into RNA by POLRMT. The primer extension reaction mixtures contained 10 nM P/T (shown at the top) and 20 nM POLRMT, and the reactions were performed in the presence of 1 μM ATP as the first ribonucleotide and increasing concentrations (in micromolar) of 3′-dCTP or NHC-TP (EIDD-2061), as indicated under each lane. The products were resolved by denaturing PAGE. The migrations of the 21-mer primer and the 22- and 23-mer first and second ribonucleotide extension products are indicated on the right. (Bottom) Quantitative analysis of nucleotide analog incorporation. The incorporation efficiency was evaluated based on the extension of 22-mer to 23-mer products. The measured *K*_1/2_ values are shown on the right of the graph. The discrimination between the analog and 3′-dCTP, D*_analog_, was calculated as *K*_1/2, analog_/*K*_1/2, 3′-dCTP_ (where *K*_1/2_ is defined as the analog triphosphate concentration resulting in 50% product extension). The D*_EIDD-2061_ in this experiment is 13.2. In second analogous experiment repeat, the D*_EIDD-2061_ was measured to be 11.5.

**FIG 3:**
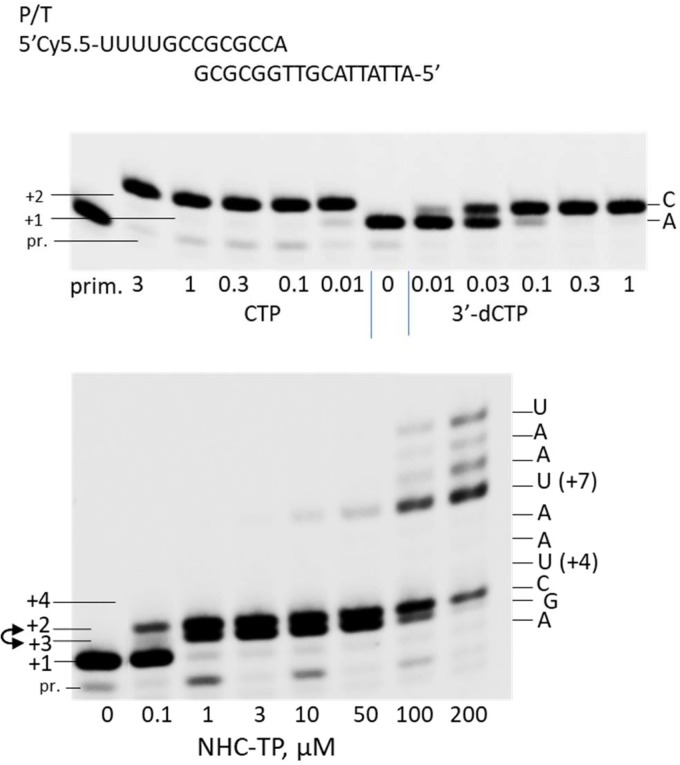
NHC-TP does not cause immediate chain termination. Primer extension reaction mixtures contained 10 nM P/T (shown at the top) and 20 nM POLRMT, and the reactions were performed in the presence of 1 μM ATP as the first ribonucleotide and increasing concentrations (in micromolar) of CTP, 3′-dCTP (top panel) or NHC-TP (bottom panel). The products were resolved by denaturing PAGE. The identity and concentration of the tested analog is indicated under each lane. The migrations of the primer (pr.) and the ribonucleotide extension products +1 to +4 are indicated on the left, and the intended nucleotide identities at each position are indicated on the right. The lane labeled “prim.” in the top panel contains unextended primer only. In the bottom panel, the location of the +2 and +3 positions are inverted due to the compression effect (indicated by a double-headed arrow).

A nonradioactive primer extension assay was used to determine whether NHC-TP can be incorporated into RNA by POLRMT as a cytidine analog. The incorporation efficiency of a ribonucleoside analog is usually compared to the natural ribonucleoside; however, in the case of CTP analogs, CTP is incorporated much more efficiently by POLRMT than most CTP analogs which makes it difficult to directly compare their incorporation efficiencies under the same conditions (7). To facilitate the measurement, 3′-dCTP can be used as an intermediary (7). In a single nucleotide incorporation primer extension assay it was determined that POLRMT favors 3′-dCTP as a substrate over NHC-TP with a discrimination value of 12.4 ± 1.2 (Fig. 2). Previously, it has been determined that POLRMT favors CTP over 3′-dCTP with a discrimination factor of 59.7 ± 1.6 (7). By extrapolation, POLRMT favors CTP as a substrate over NHC-TP by a factor of approximately 740. This value suggests that NHC-TP is a relatively efficient substrate for POLRMT (7). It was further determined that NHC-TP does not cause immediate chain-termination upon incorporation into nascent chain RNA in an alternative assay where multiple nucleotides could be incorporated (Fig. 3). As shown in Fig. 3, after NHC-TP was incorporated as a C-analog at the +2 position, NHC-TP can be further incorporated as a G-analog forming a +3 band and as a U-analog at the +4 position in conditions when no competing natural GTP or UTP are present. Partial chain termination occurs at multiple sites further downstream after incorporation of NHC-TP, as demonstrated by accumulation of the unextended bands at the +7 to +9 positions.

### NHC causes stronger reduction in mitochondrial DNA-encoded protein expression than in nuclear DNA-encoded protein expression.

**FIG 4:**
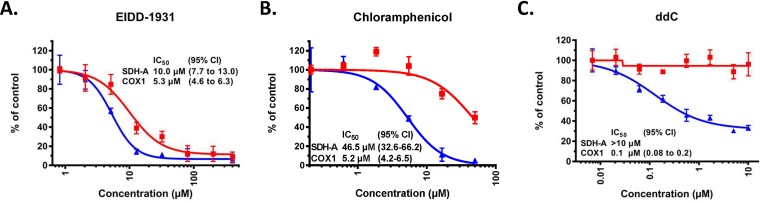
Representative experiment showing dose-response plots of NHC (A), chloramphenicol (B), and ddC (C) inhibition of COX1 and SDH-A protein expression in PC-3 cells. COX1 protein levels are indicated by blue triangles, and SDH-A protein levels are indicated by red squares. IC_50_ values are listed in μM with the 95% confidence intervals listed in parentheses. Data were generated using Prism 8 software, and each value shown is the average ± the SD (*n* = 3).

Since NHC-TP is a substrate for POLRMT and the NHC-modified RNA may have different translation properties, it is important to understand if POLRMT-mediated protein expression is selectively affected in cells incubated with NHC. The effect of NHC on expression of mitochondrial DNA-encoded protein cytochrome *c* oxidase subunit 1 (COX1) was compared to the effect on expression of nuclear DNA-encoded protein succinate dehydrogenase A (SDH-A) in PC-3 cells using an enzyme-linked immunosorbent assay (ELISA)-based quantitative assay. PC-3 cells were chosen because they have been shown to be highly sensitive to mitochondrial toxins in this assay and, therefore, are preferable for such analysis (22). The results of this assay are shown in Fig. 4 and Table 2. In PC-3 cells incubated with increasing concentrations of NHC for 5-days, the measured 50% inhibitory concentration (IC_50_) value for COX1 protein expression was 2.7-fold lower relative to the IC_50_ for SDH-A protein expression, suggesting that NHC has slightly stronger negative effect on mitochondrial mRNA transcription and/or translation. For comparison, chloramphenicol, which was used as a positive control, exhibited stronger effect on mitochondrial protein expression, with IC_50_ values of COX1 protein inhibition ∼12-fold lower compared to that of SDH-A protein (see Table S2). Similarly, dideoxycytidine (ddC) showed selective inhibition of COX1 expression with an SDH-A/COX1 IC_50_ ratio of >49.2.

**TABLE 2:** NHC inhibition of protein expression of COX1 and SDH-A^*a*^

Expt	IC_50_ (μM)		SDH-A/COX1 ratio
	COX1	SDH-A	
1	5.3	10.0	1.9
2	6.5	24.8	3.8
3	7.7	15.4	2.0
4	5.3	16.7	3.2

a NIC inhibition values were calculated by four-parameter curve fit by using GraphPad Prism 8 software. The results from four individual experiments are shown. The averages ± the standard deviations for COX1 and SDH-A (IC_50_ [μM]) and for the SDH-A/COX1 ratio were 6.2 ± 1.1, 16.7 ± 6.1, and 2.7 ± 0.9, respectively.

### Long-term incubation with NHC in CEM or HepG2 cells does not cause a reduction in mtDNA levels, or an increase in lactate production.

Mitochondrial toxicity can be exhibited by a decrease in mitochondrial number per cell or by a decrease of function in existing mitochondria. The decrease of functional activity of mitochondria should result in less production of ATP by mitochondria even if the mitochondrial number has not changed. In glucose-containing culture medium conditions, the decrease in mitochondrial number or in mitochondrial function in the affected cell puts additional demands on ATP production through mitochondrion-independent anaerobic glycolysis, which is registered by an increase in lactate production. To determine whether any of the mechanisms are involved, i.e., the decrease in mitochondrial quantity or the decrease of mitochondrial function due to decrease in mitochondrial protein quantity or protein function (13, 21, 23), the effect of long-term incubation with NHC on both mitochondrial number and lactate production in treated cells was investigated.

To measure the long-term effects of exposure to NHC, HepG2 cells were incubated with NHC at 10 and 100 μM for 14 days, and CEM cells were incubated with NHC at 10 and 30 μM for 14 days. The concentrations were selected near or just above the CC_50_ concentrations for the corresponding cell line. Higher treatment concentrations could not be tested since not enough cells survived to perform the assay. After 14 days of treatment, mtDNA copy number in cells and lactate levels in cell culture media were measured relative to the mock-treated cells. ddC, a known inhibitor of DNA polymerase γ (24), and ethidium bromide (EtBr), a mitochondrial replication inhibitor (25), were used as positive controls. Data from these experiments are summarized in Tables 3 and 4. There was not a significant difference in relative mtDNA levels in HepG2 cells treated with NHC for 14 days at either concentration. After treatment at 10 μM, the level of relative mtDNA in HepG2 cells was 115.0 ± 92.4% compared to a mock-treated control, and after treatment at 100 μM, relative level of mtDNA was 68.4 ± 17.6% of the control, but the difference was not statistically significant (*P* = 0.8). Parallel treatment with positive controls ddC and EtBr resulted in mtDNA decreases to 7.1 ± 2.8% and 19.6 ± 12.1%, respectively, compared to a mock-treated control. The lactate levels in the NHC-treated cells were 103.7 ± 21.2% relative to the mock-control when cells were treated at 10 μM and 111.5 ± 38.5% when treated at 100 μM, indicating no significant increase in lactate production after 14 days of treatment at either concentration. Treatment with positive controls ddC and EtBr resulted in extracellular lactate increases to 195.9 ± 61.9% and 242.5 ± 47.8%, respectively. In CEM cells treated with NHC at 10 or 30 μM, relative mtDNA was measured at 80.0 ± 19.1% and 78.7 ± 15.8%, respectively, compared to the mock-treated controls (100 ± 10.8%). Although the differences of relative mtDNA levels from the controls were statistically significant (*P* < 0.05), they were likely not meaningful, particularly considering that no additional decrease was observed at a higher treatment concentration. For comparison, in CEM cells treated with positive controls, the relative mtDNA levels were decreased to 0.15 ± 0.06% after incubation with ddC and to 0.39 ± 0.11% for cells incubated with EtBr. The lactate levels in CEM cells incubated with NHC at 10 μM were increased slightly to 118.1 ± 3.4% relative to the mock control but were not increased after incubation with NHC at 30 μM (101.8 ± 12.7%). In CEM cells treated with positive controls, the lactate levels were 310.5 ± 71.2% after treatment with ddC and 260.7 ± 39.3% after treatment with EtBr.

**TABLE 3:** Relative mtDNA and extracellular lactate levels in HepG2 cells after a 14-day incubation with various compounds

Treatment compound	Treatment concn	Mean ± SD^*a*^	
		Relative mtDNA level	Lactate production
Mock-treated control	NA^*b*^	100 ± 25.8	100
NHC	10 μM	115.0 ± 92.4	103.7 ± 21.2
	100 μM	68.4 ± 17.6	111.5 ± 38.5
ddC	1 μM	7.1 ± 2.8*	195.9 ± 61.9
EtBr	0.5 μg/ml	19.6 ± 12.1	242.5 ± 47.8**

a Values are means from three independent experiments (with one biological and three technical repeats each). *, *P* < 0.05; **, *P* < 0.01 (compared to a mock-treated control).

b NA, not applicable.

**TABLE 4:** Relative mtDNA and extracellular lactate levels in CEM cells after a 14-day incubation with various compounds

Treatment compound	Treatment concn	Mean ± SD^*a*^	
		Relative mtDNA level	Lactate production
Mock-treated control	NA^*b*^	100 ± 10.8	100 ± 0
NHC	10 μM	80.0 ± 19.1*	118.1 ± 3.4**
	30 μM	78.7 ± 15.8*	101.8 ± 12.7
ddC	0.2 μM	0.15 ± 0.06***	310.5 ± 71.2**
EtBr	0.1 μg/ml	0.39 ± 0.11***	260.7 ± 39.3**

a Values are means from three independent experiments (with one biological and three technical repeats each). *, *P* < 0.05; **, *P* < 0.01; ***, *P* < 0.001 (compared to a mock-treated control).

b NA, not applicable.

## DISCUSSION

*N*^4^-hydroxycytidine, a ribonucleoside analog, is currently in late stage, preclinical development as a broad-spectrum antiviral agent. Since a number of nucleoside analogs have been halted at different stages of development due to mitochondrial toxicity (2, 8–10), we conducted an investigation to determine the potential for NHC to interact with cellular processes that could lead to mitochondrial toxicity and dysfunction. A previously reported study has concluded that NHC does not cause mitochondrial toxicity based on the observation that NHC has no effect on mitochondrial DNA or RNA levels after a 7-day treatment (14). Here, we report on a more comprehensive series of experiments designed to thoroughly investigate the potential for NHC to cause mitochondrial toxicity over a longer treatment period, at higher concentrations, and in cell lines that are most sensitive to the cytotoxicity of NHC.

The results indicate that mitochondrial impairment by NHC does not substantially contribute to the observed cytotoxicity. In glucose-free media, where a mitochondrial toxicant can have a more significant impact due to the Crabtree effect, no differences were observed in the cytotoxicity CC_50_ value compared to the CC_50_ observed in glucose-containing media. While NHC–5′-triphosphate does not inhibit DNA-polymerases α, β, and γ at 1,000 μM (Table S3), incorporation into cellular RNA could be a cause of observed cytotoxicity. Incorporation of NHC residues into Pol II RNA could be suggested from an elevated level of mutations found in Pol II-transcribed mRNA in the presence of NHC (26). Cellular RNA Pol II possesses a safeguard activity from misincorporation and is able to excise misincorporated nucleotides (27). However, POLRMT lacks this ability (28), and given that NHC is an efficient substrate for POLRMT, it has the potential to disrupt vital mitochondrial functions such as respiration-dependent ATP production (8). POLRMT-synthesized short RNAs serve as primers in mitochondrial DNA synthesis by DNA polymerase γ, and inhibition of mitochondrial RNA synthesis as a result of NHC incorporation could potentially affect the replication of mtDNA (29). NHC caused partial, delayed chain termination after incorporation by POLRMT, which could potentially cause toxicity due to a decrease in the quantity of mitochondrial RNA. On the other hand, high concentrations of competing CTP, UTP, and GTP present in the cell at physiological concentrations ranging from 200 to 600 μM (30) may reduce the likelihood that NHC is misincorporated to a level that would affect RNA synthesis. Stuyver et al. showed that mitochondrial mRNA levels were unchanged in HepG2 cells incubated with NHC at 10 μM for 7 days (14), suggesting that NHC-TP is not incorporated at the frequency required to reduce mitochondrial RNA synthesis. NHC was incorporated by POLRMT as a cytidine and uridine analog and, to a lesser extent, as a guanosine analog. Incorporation of NHC as both cytidine and uridine analogs may result in the production of mutagenized mitochondrial mRNAs that are either translated less efficiently or translated into mutated and potentially less-functional proteins. In fact, mutations in nuclear and mitochondrial mRNA were observed in tissue culture cells that were treated with NHC at 10 μM for 3 days; however, there was no increase in the frequency of mitochondrial or nuclear mRNA mutations in ferrets that were given seven doses of NHC twice daily (b.i.d.) at 200 mg/kg/day, despite the fact that plasma levels of NHC were expected to be above 10 μM at any time throughout the treatment course (26). It has been shown that NHC was efficiently anabolized to the triphosphate form in primary hepatocytes and in animal organs *in vivo* (16, 26, 31). The decrease in quantity of mitochondrial protein or the potential decrease of functional activity of mitochondrial proteins may result in less production of ATP by the mitochondria even if the mitochondrial number has not changed. If this happens, the affected cell switches its ATP production to mitochondrion-independent anaerobic glycolysis, which can be registered by an increase in lactate production. Although, at some concentrations, there were differences between mitochondrial and nuclear protein expression (see Fig. 4A), these differences are not translated into a decrease in number of mitochondria per cell (as measured by mtDNA/cell), or in an increase in lactate production after a long incubation with NHC. In HepG2 cells treated with NHC for 14 days, there was not a significant difference in mtDNA levels or extracellular lactate levels. In CEM cells, relative mtDNA levels were reduced by about 20% at both 10 and 30 μM concentrations of NHC. Extracellular lactate levels also increased by less than 20% in the cells incubated with the lower concentration, but there was no change in extracellular lactate levels in cells incubated with the higher concentration of NHC. It has been previously suggested that increases in extracellular lactate of less than 20% are not biologically relevant (32). The registered effects in CEM cells were likely not indicative of mitochondrial toxicity since there was no dose response in relative mtDNA depletion and there was no increase in extracellular lactate at the higher NHC concentration.

It has been suggested that there is a spare capacity in mitochondria to tolerate a reduction in respiratory chain complex activity before there is an impact on ATP synthesis or mitochondrial respiration (33). It was shown that the activity of cytochrome *c* oxidase, a protein in the mitochondrial respiratory chain complex, in mitochondria isolated from rat muscle and brain could be inhibited by 70 to 80% with KCN without any major changes in mitochondrial respiration or ATP synthesis (34, 35). It has also been shown that in cells containing transplanted mtDNA that contained stop codons for the COX1 gene in 85% of its mtDNA, COX1 activity was still measured at 70% relative to cells that contained all nonmutant mtDNA (33). It has been theorized that the existing excess of respiratory chain complexes allows for a “threshold effect,” providing mitochondria a spare amount of enzymes necessary to function even when some enzymes have been diminished up to a point (36). It has been confirmed that there is an excess capacity of cytochrome *c* oxidase for respiration in mitochondria (37), which further suggested that this could be the mechanism for the threshold effect.

NHC inhibits the expression of mitochondrial DNA encoded COX1 protein at lower concentrations compared to expression of nuclear DNA encoded SDH-A protein in PC-3 cells. The IC_50_ for COX1 protein expression is 2.7-fold lower than the IC_50_ for SDH-A protein expression, which suggests that NHC has a stronger impact on COX1 protein expression, possibly due to the absence of proofreading activity for POLRMT. There has been little discussion on the relevance of these differences in publications. On the other hand, the measured CC_50_ for NHC in PC-3 cells, which were based on the quantitation of intracellular ATP levels, was determined to be 261.7 μM (see Table 1 and Fig. S1), which suggests that treatment with NHC up to ∼100 μM does not inhibit mitochondrial function above the threshold level necessary to affect ATP synthesis. Current *in vivo* treatment data with NHC also indicate that there is significant tolerability of NHC in animals, at least for short-duration treatments. It has been reported that NHC is well tolerated in mice and guinea pigs that were given b.i.d. oral doses at 800 mg/kg/day for 6 days in mice and for 3 days in guinea pigs (16). NHC was also well tolerated in ferrets given b.i.d. oral doses of NHC at 200 mg/kg/day for 3.5 days (26). Another group has reported that six daily doses by intraperitoneal (i.p.) injection of NHC at 33 mg/kg/day were also well tolerated in mice, though an elevated i.p. dose of NHC at 100 mg/kg/day apparently caused a weight loss, which was reversible immediately after the end of dosing (14). This quick reversibility would imply that there was no permanent damage to mitochondrial function in any organ.

The 5′-isopropyl ester prodrug of *N*^4^-hydroxycytidine, designated EIDD-2801 (26), is currently under development for the treatment of a broad range of RNA virus infections. The initial focus will be on the treatment of encephalitic alphavirus infections and influenza. Although extensive work in animal models of both infections indicate the duration of treatment will be short, most likely between three to 5 days of dosing, every effort is being made to determine whether mitochondrial toxicity could still impact utility. To that end, the studies reported here were extended for up to 14 days using very high concentrations of NHC. The results strongly suggest that mitochondrial toxicity will not be a dose or duration of treatment limiting issue. Chronic-toxicity studies (28 days) are under way in rats and dogs, and every effort will be made to monitor for toxicities arising from mitochondrial dysfunction.

## MATERIALS AND METHODS

### Compounds and chemicals.

*N*^4^-Hydroxycytidine (NHC; EIDD-1931) was prepared as previously described (16). ddC was purchased from Tokyo Chemicals Industry America (Boston, MA), and ethidium bromide was from Sigma-Aldrich (St. Louis, MO). All other chemicals were from commercial sources.

### Cell lines.

HepG2 cells (ATCC) for use in cytotoxicity assays (HepG2/Gal) were maintained for three passages in glucose-free RPMI 1640 media (VWR International, Suwanee, GA) supplemented with 7.5 mM galactose (Sigma-Aldrich), 10% fetal bovine serum (FBS; Atlanta Biological, Flowery Branch, GA), 2 mM l-glutamine (VWR), and 1× penicillin-streptomycin (VWR). HepG2 cells for use in other assays were maintained in glucose-containing RPMI 1640 (VWR) supplemented with FBS, 2 mM l-glutamine, and 1× penicillin-streptomycin. CEM cells (ATCC) were maintained in RPMI 1640 media supplemented with 10% FBS, 2 mM l-glutamine and 1× penicillin-streptomycin. PC-3 cells (ATCC CRL-1435) were incubated in Ham’s F-12K complete medium (Thermo Fisher Scientific, Waltham, MA) containing 10% FBS HI, 2 mM l-glutamine, and 1× penicillin-streptomycin.

### Cytotoxicity of NHC in mammalian cell lines.

HepG2/Gal cells were divided into two parts; the first was resuspended and seeded in glucose-free media, and the second was resuspended in glucose-containing media. HepG2 cells and PC-3 cells were plated in 96-well plates at 1 × 10^4^ cells per well, followed by incubation overnight for attachment before addition of drug. CEM cells were plated at 2 × 10^4^ cells per well immediately before drug addition. Cells were incubated in the presence of increasing concentrations of NHC from 0.8 to 400 μM for 3 days at 37°C in a 5% CO_2_ atmosphere. Cell viability, as a measurement of intracellular ATP levels, was determined using the CellTiter-Glo cell viability assay kit (Promega, Madison, WI) according to the manufacturer’s instructions.

### Uptake and anabolism of NHC in HepG2 and CEM cell lines.

HepG2 cells, PC-3 cells, and CEM cells were incubated with NHC as previously described (16). Briefly, HepG2 cells were incubated with NHC at 20 μM for 0, 1, 2, 3, 4, 6, 16, and 24 h and then washed with phosphate-buffered saline (PBS) and extracted with 70% methanol in water. PC-3 cells were incubated with NHC at 10 μM for 0, 1, 2, 3, 4, 6, 16, and 24 h and then washed with PBS and extracted with 70% acetonitrile in water. Extracts were centrifuged and stored at −20°C until analysis by LC-MS/MS. CEM cells were incubated with NHC at 10 μM for 0, 1, 3, 6, and 24 h. The cells were centrifuged and washed with PBS, and then the cell pellets were extracted with 70% methanol in water. Extracts were clarified by centrifugation and stored at −20°C until analysis. The 5′-triphosphate metabolite of NHC (NHC-TP) was quantitated by LC-MS/MS with an Agilent 1200 system (Agilent Technologies, Santa Clara, CA) equipped with a SeQuant ZIC-pHILIC column (Millipore/Sigma, Burlington, MA). Mobile phase A consisted of 25 mM ammonium bicarbonate in water at pH 9.8, and acetonitrile was used as mobile phase B. Mass spectrometry analysis was performed on a QTrap 5500 mass spectrometer (Sciex, Framingham, MA) in multiple-reaction-monitoring mode. Data analysis was performed using Analyst software (Sciex, Framingham, MA).

### POLRMT primer extension assay.

POLRMT primer extension reactions were performed as previously described (7). Briefly, a 10 nM concentration of a fluorescently labeled RNA primer annealed to a DNA template (primer/template [P/T]; the sequences are shown at the top of corresponding figures), 20 nM POLRMT, 1 μM ATP, and increasing concentrations of 3′-dCTP or NHC–5′-triphosphate (NHC-TP) were incubated at 22°C for 30 min; the samples were then separated using denaturing polyacrylamide gel electrophoresis (PAGE). Gels were scanned using an Odyssey infrared imaging system (LI-COR Biosciences, Lincoln, NE), and the proper RNA bands were quantified using the Image Studio Lite software (v4.0; Li-COR Biosciences).

### Protein expression.

PC-3 cells were treated with test compounds at increasing concentrations for 5 days and analyzed as previously described with a colorimetric MitoBiogenesis In-Cell ELISA kit (Abcam, Cambridge, United Kingdom) (12). Briefly, PC-3 cells were plated in 96-well plates at a density of 2.5 × 10^3^ cells/well in Ham’s F-12K complete medium. After 5 days of incubation with drug, the cellular levels of the mitochondrial DNA-encoded protein, COX1, and the nuclear DNA-encoded protein, SDH-A, were analyzed using the assay kit according to the manufacturer’s protocol. Colorimetric analysis was performed using a SpectraMax M2 spectrophotometer (Molecular Devices, San Jose, CA). Chloramphenicol and ddC were used as positive controls.

### Quantification of mtDNA and lactate levels.

HepG2 cells were treated in sodium pyruvate-containing Dulbecco modified Eagle medium supplemented with 10% dialyzed FBS (Life Technologies), 50 μM uridine (Alfa Aesar, Haverhill, MA), l-glutamine, and penicillin-streptomycin. Cells were seeded at 4 × 10^4^ cells/well in 2 ml of media per well in 12-well plates 1 day before the addition of the test compounds. CEM cells were treated in RPMI 1640 media supplemented with 10% dialyzed FBS, 1 mM sodium pyruvate (Life Technologies), 50 μM uridine (Alfa Aesar), l-glutamine, and penicillin-streptomycin. CEM cells were seeded at 2 × 10^4^ cells/ml in 2 ml of media per well in 12-well plates on the day of the addition of drug. Cells were cultured continuously in 5% CO_2_ for 14 days in the presence of compounds. For HepG2 cells, the medium was changed on day 6 and day 9 to media with fresh drug. CEM cells were passaged by 1:10 dilution on days 6 and 9 in media with fresh drug. On day 14, the cell-free culture medium was collected. Cells were collected and counted using disposable hemocytometers (INCYTO, Covington, GA). Each experiment was repeated three times with each repeat initiated on different days. The lactate concentration in cell-free medium was measured by using an EnzyChrom l-lactate assay kit (BioAssay System, Hayward, CA). Briefly, all of the samples were diluted 20-fold with water, and the lactate concentration was measured according to the manufacturer’s instructions. The lactate concentration was expressed as a percentage of the mock-treated controls. The effect of drugs on mitochondrial DNA quantity per cell was measured by comparing the relative mitochondrial genome copy number (mtDNA copy number divided by nuclear DNA copy number) in cells treated with drug versus mock-treated cells. A nuclear DNA target sequence was used that corresponds to the β-actin gene (38), and a mitochondrial target sequence was used that corresponds to mitochondrial DNA nucleotide positions 10620 to 10710 (39). The primers and TaqMan probes (Eurofins MWG Operon, Louisville, KY) used for real-time PCR were ordered were as follows. The primers and probe used for the quantification of nuclear DNA (β-actin gene) were the sense primer 5′-GCGCGGCTACAGCTTCA-3′, the antisense primer 5′-TCTCCTTAATGTCACGCACGAT-3′, and the probe 5′-(FAM)-CACCACGGCCGAGCGGGA-(BHQ)-3′. The primers and probe for the quantification of mtDNA were the forward primer MH533 (5′-ACCCACTCCCTCTTAGCCAATATT-3′), the reverse primer MH534 (5′-GTAGGGCTAGGCCCACCG-3′), and Mito-Probe [5′-(FAM)-CTAGTCTTTGCCGCCTGCGAAGCA-(BHQ)-3′]. The cells were collected after 14 days of drug treatment and pelleted by centrifugation at 8,600 × *g* for 2 min. The cell pellets were resuspended in PBS, and the total DNA was isolated by using a DNeasy tissue kit (Qiagen, Hilden, Germany). Cellular DNA from mock-treated cells was serially diluted and used to generate corresponding standard curves for determining a relative copy number of the gene targets. qPCR of each sample was performed in triplicate in a 7500 real-time PCR system (Applied Biosystems, Foster City, CA) in a 15-μl total reaction volume containing 1× PCR mix (Life Technologies, Carlsbad, CA), 200 nM β-actin probe (for nuclear DNA) or Mito-Probe (for mtDNA), 750 nM β-actin sense and antisense primers (for nuclear DNA) or MH533/MH534 primers (for mtDNA), and 1.5 μl of a purified, serially diluted DNA sample. The mitochondrial and cellular genome copy numbers were calculated based on the standard curves, and the relative mitochondrial copy number/cell was calculated by dividing the measured mitochondrial copy number by the copy number of the β-actin gene. Changes in the mitochondrial copy number/cell were expressed as a percentage of the mock-treated control.

## Supplementary Material

Supplemental file 1
