# Supplementary material for: Analysis of the Potential for N4-Hydroxycytidine To Inhibit Mitochondrial Replication and Function
Source: Antimicrob Agents Chemother. 2020 Jan 27;64(2):e01719-19. doi: 10.1128/AAC.01719-19 (PMC6985706; doi:10.1128/AAC.01719-19)
Supplement: Supplemental file 1 [file AAC.01719-19-s0001.pdf]

Supplemental Material

Supplemental Table S1. CC_{50} values of NHC in various mammalian cell lines

Cell Line	A204	A549	BxPC-3	Huh-7	H9c-2	Vero	HEp-2	MDCK
CC_{50} (µM)	84	46	48	165.5	81	53	272.4[1]	299.8[1]

[1] from Yoon et al., 2018

Supplemental Text S1: Cell Lines and Cytotoxicity assays

A-204 cells (ATCC HTB-82) were incubated in McCoys Complete medium (McCoy's 5a Medium, 10% Fetal Bovine Serum, Heat Inactivated (FBS HI), 2 mM L-Glutamine, and 1 X Penicillin/Streptomycin). BxPC-3 cells (ATCC CRL-1687) were incubated in RPMI 1640 Complete Media (RPMI 1640, 10% FBS HI, 2 mM L-Glutamine, and 1 X Penicillin/Streptomycin). H9c2 cells (ATCC CRL-2446), Huh-7 cells (Japan Health Sciences Foundation, Osaka, Japan) and A549 cells (from Richard Plemper's lab, Georgia State University, Atlanta, GA), were incubated in DMEM Complete Media (Dulbecco Modified Essential Medium, 10% FBS HI, 2 mM L-Glutamine, and 1 X Penicillin/Streptomycin). IEC-6 cells (ATCC CRL-1592) were incubated in DMEM Complete Media supplemented with 0.1 Unit/mL bovine insulin. Vero cells (ATCC CRL-81) were incubated in MEM Complete Media (MEM, 10% FBS HI, 2 mM L-Glutamine, and 1 X Penicillin/Streptomycin).

The day preceding the assay, cell lines were trypsinized and total cell count and viability quantification was performed using a hemocytometer and Trypan Blue (Fisher, Suwanee, GA) dye exclusion method. For analysis of cytotoxicity utilizing the CellTiter-Glo Luminescent Cell Viability Assay (Promega, Madison, WI), H9c-2 cells were resuspended at 5 x 10^3 cells per 50 µl in corresponding complete media and all other cell lines were resuspended at 10 x 10^3 cells per 50 µl. Cells were seeded in white 96-well Nunclon™ white microtiter plates (Fisher, Suwanee, GA) in a volume of 50 µl/well. The plates were incubated at 37°C/5% CO_2 overnight to allow for cell adherence.

Plate Format: Each plate contained cell control wells (cells only) and drug treatment wells (cells plus drug) in triplicate for each drug dilution. Fifty microliters of the drug dilution at 2X final concentration was added to the appropriate cell wells. The final drug concentration was 400, 160, 64, 25.6, 10.24, 4.1, 1.64 and 0.66 µM (2.5X dilutions). Media without drug was added to the cell control wells.

For the CellTiter-Glo luminescent cell viability assay, cells were incubated with NHC at 37°C in a 5% CO_2 incubator for 3 days. Following incubation, the media and drug was discarded from the plates. The substrate was diluted 1:1 with Dulbecco's Phosphate Buffered Saline (DPBS) and 30 µl of the diluted substrate was added to each well. The plates were incubated at room temperature for 10 minutes to stabilize luminescence signal. The plates were read in a Veritas Microplate Luminometer (Turner Biosystems, Mountain View, CA).

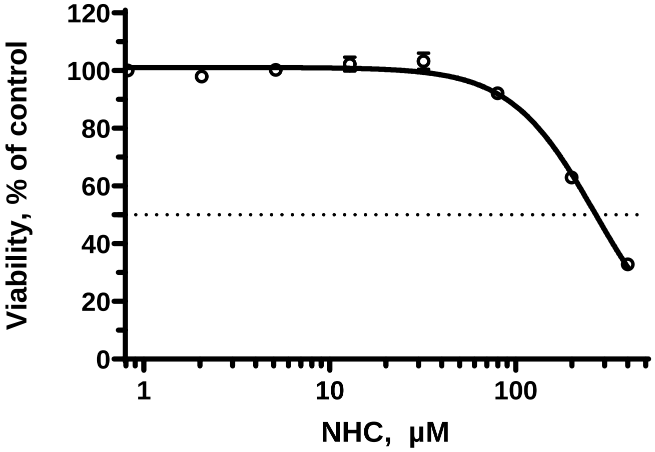

Supplemental Figure S1. Cytotoxicity of NHC in PC-3 cells after a 3 day incubation. Values are shown as Mean ± SD (n=3). The calculated CC_{50} is 267.1 µM calculated with GraphPad Prism 8 software.

Supplemental Table S2. Effects of Chloramphenicol and ddC on Protein expression

Compound	IC_{50} COX1 (μM)	IC_{50} SDH-A (μM)	SDH-A/COX1 ratio
**Chloramphenicol**[1]	0.30	3.0	10.0
	4.7	36.3	7.7
	5.2	46.5	8.9
	4.7	>100	>21.4
**Average**	**3.7**	**46.4**	**12.0**
**SD**	**2.3**	**40.3**	**6.4**
**ddC**[1]	0.12	>10	>83.5
	6.7	>10	>1.5
	0.16	>10	>62.4
**Average**	**2.3**	**>10**	**>49.2**
**SD**	**3.8**	**0**	**42.6**

[1] each pair of COX1/SDH-A values represent individual experiments with n=3 biological repeats in each experiment

Supplemental Table S3. Inhibition of DNA polymerases α, ß, and γ by EIDD-2061

DNA Polymerase	IC_{50} (µM)
DNA pol α	>1000
DNA pol ß	>1000
DNA pol γ	>1000

Supplemental Text S2: DNA polymerase Inhibition Assays

The human DNA polymerases α, ß, and γ were purchased from CHIMERx (Madison, WI). Inhibition of DNA polymerase ß and γ activity was assayed in microtiter plates in a 50 µl reaction mixture containing 50 mM Tris-HCI (pH 8.7), KCl (10 mM for ß and 100 mM for γ), 10 mM $MgCl_2$, 0.4 mg/ml BSA, 1 mM DTT, 15% glycerol, 0.05 mM of dCTP, dTTP, and dATP, 10 µCi [^{32}P]-α-dGTP (800 Ci/mmol), 20 µg activated calf thymus DNA and increasing comcentrations of EIDD-2061 (NHC-TP). The DNA polymerase α reaction mixture was as follows in a 50 µl volume per sample: 20 mM Tris-HCl (pH 8), 5 mM Mg acetate, 0.3 mg/ml BSA, 1 mM DTT, 0.1 mM spermine, 0.05 mM of dCTP, dTTP, and dATP, 10 µCi [^{32}P]-α-dGTP (800 Ci/mmol), 20 µg activated calf thymus DNA and the test compound at increasing concentrations. For each assay, the enzyme reactions were allowed to proceed for 30 min at 37°C followed by the transfer onto glass-fiber filters and subsequent precipitation with 10% trichloroacetic acid (TCA). The filter was then washed five times with 5% TCA followed by one wash with 95% ethanol and air-drying. Once the filter had dried, incorporation of [α-^{32}P] GTP was measured using a liquid scintillation counter (Microbeta). Raw data was collected from the scintillation counter and imported into a Microsoft Excel 2007 spreadsheet for analysis by linear curve fit calculations.

Supplemental References

1. Yoon JJ, Toots M, Lee S, Lee ME, Ludeke B, Luczo JM, Ganti K, Cox RM, Sticher ZM, Edpuganti V, Mitchell DG, Lockwood MA, Kolykhalov AA, Greninger AL, Moore ML, Painter GR, Lowen AC, Tompkins SM, Fearns R, Natchus MG, Plemper RK. 2018. Orally Efficacious Broad-Spectrum Ribonucleoside Analog Inhibitor of Influenza and Respiratory Syncytial Viruses. Antimicrob Agents Chemother 62.
